# AraCust: a Saudi Telecom Tweets corpus for sentiment analysis

**DOI:** 10.7717/peerj-cs.510

**Published:** 2021-05-20

**Authors:** Latifah Almuqren, Alexandra Cristea

**Affiliations:** 1 Department of Computer Science, Durham University, Durham, United Kingdom; 2 Information Science Department, Computer and Information Sciences College, Princess Nourah bint Abdulrahman University, Riyadh, Saudi Arabia

**Keywords:** Sentiment analysis, Arabic, Gold Standard Corpus, Supervised approach

## Abstract

Comparing Arabic to other languages, Arabic lacks large corpora for Natural Language Processing (Assiri, Emam & Al-Dossari, 2018; Gamal et al., 2019). A number of scholars depended on translation from one language to another to construct their corpus (Rushdi-Saleh et al., 2011). This paper presents how we have constructed, cleaned, pre-processed, and annotated our 20,0000 Gold Standard Corpus (GSC) AraCust, the first Telecom GSC for Arabic Sentiment Analysis (ASA) for Dialectal Arabic (DA). AraCust contains Saudi dialect tweets, processed from a self-collected Arabic tweets dataset and has been annotated for sentiment analysis, i.e.,manually labelled (k=0.60). In addition, we have illustrated AraCust’s power, by performing an exploratory data analysis, to analyse the features that were sourced from the nature of our corpus, to assist with choosing the right ASA methods for it. To evaluate our Golden Standard corpus AraCust, we have first applied a simple experiment, using a supervised classifier, to offer benchmark outcomes for forthcoming works. In addition, we have applied the same supervised classifier on a publicly available Arabic dataset created from Twitter, ASTD (Nabil, Aly & Atiya, 2015). The result shows that our dataset AraCust outperforms the ASTD result with 91% accuracy and 89% F1avg score. The AraCust corpus will be released, together with code useful for its exploration, via GitHub as a part of this submission.

## Introduction

With the growing use of social media sites worldwide over the last ten years, sentiment analysis (SA) has recently become a prominent and useful technique for capturing public opinion in many different disciplines. SA, or “opinion mining,” refers to a computational process of gathering individuals’ opinions, feelings, or attitudes towards a particular event or issue (Abdulla et al., 2014; Al-Thubaity et al., 2018). SA has a vital function in real-life applications and decision-making processes in various domains (Al-Twairesh et al., 2017; Al-Twairesh & Al-Negheimish, 2019).

The detection of sentiment polarity, however, is a challenging task, due to limitations of sentiment resources in different languages. While a substantial body of research exists for English (Assiri, Emam & Al-Dossari, 2018; Al-Twairesh, 2016) it remains a largely unexplored research area for the Arabic language (Assiri, Emam & Al-Dossari, 2018; Al-Twairesh, 2016; Al-Twairesh et al., 2017; Habash, 2010), even though there is an enormous population of Arabic speakers (274 million worldwide in 2019 Eberhard, Gary & Fennig (2021); 5th in the world). This is due chiefly to the complexity of Arabic (Habash, 2010; Al-Twairesh, 2016; Al-Twairesh et al., 2018b). It has many forms, including *Classical Arabic (CA)*, as in the book of Islam’s Holy Quran, *Modern Standard Arabic (MSA)* used in newspapers, education, and formal speech, and *Dialectical Arabic (DA),* which is the informal everyday spoken language, found in chat rooms and social media platforms. The Arabic language consists of 28 Arabic alphabet letters, eight of which come in two forms (Habash, 2010). Diacritics are used, which are small marks over or under letters positioned to reflect vowels. DA forms differ from one Arab country to another. Mubarak & Darwish (2014) defined six Arabic dialects: Gulf, Yemeni, Iraqi, Egyptian, Levantine, and Maghrebi.

In 2020, Saudi Arabia reached 12 million Twitter users (Statista, 2020). But for the Gulf dialect, especially the Saudi dialect, fewer Saudi dialect corpus and lexicon resources exist than for other Arabic dialects. For instance, the Egyptian dialect has had a lot of attention, as has Levantine Arabic (Al-Twairesh, 2016). Current efforts have concentrated on the Gulf dialect (Khalifa et al., 2016a) and the Palestinian dialect (Jarrar et al., 2017), but resources used for one Arabic country cannot be applied to another. Thus, there is still a need for Arabic corpora, including DA (El-Khair, 2016); especially pressing is the need to incorporate Saudi DA (Al-Twairesh, 2016).

There is also a lack of DA datasets and lexicons, especially freely available GSC Saudi datasets (Assiri, Emam & Al-Dossari, 2018). Unfortunately, the availability of the few existing resources is limited, due in part to strict procedures for gaining permission to reuse aggregated data, with most existing corpora not offering free access. Additionally, DA analysis, targeted here, is complicated, requiring a native speaker.

Finally, the telecom field has changed with the emergence of new technologies. This is also the case with the telecom market in Saudi Arabia, which expanded in 2003 by attracting new investors. As a result, the Saudi telecom market became a viable market (Al-Jazira, 2020).

This research aims to fill these gaps, by creating a gold-standard Saudi corpus AraCust and Saudi lexicon AraTweet for use in data mining, specific to the telecom industry.

This paper’s main contributions are as follows. It focuses on Arabic Sentiment Analysis and provides solutions to one of the challenges that faces Arabic SA by creating the largest *Saudi GSC*. This resource is based on data extracted from Twitter. It is also the first corpus specifically targeted to the telecom sector. It also provides an evaluation of this corpus, further demonstrating its quality and applicability.

First, we review related research. Then, the methodology that was used in this research to build the gold-standard annotation corpus is presented. Next, it provides the corpus validation. Finally, conclusions are drawn.

## Related Research

Compared to other languages, Arabic lacks a large corpus (Assiri, Emam & Al-Dossari, 2018; Al-Twairesh, 2016; Al-Twairesh et al., 2017; Habash, 2010; Gamal et al., 2019). Many scholars have depended on translation from one language to another to construct their corpus. For example, the Opinion Corpus for Arabic (OCA), one of the oldest and most-used corpora for ASA (Rushdi-Saleh et al., 2011), is comprised of more than 500 Arabic movie reviews. The reviews were translated by automatic machine translation, and the results compared to both Arabic and English versions. Subsequently, most research efforts have focused on enhancing classification accuracy with the OCA dataset (Atia & Shaalan, 2015). In addition, the MADAR (http://nlp.qatar.cmu.edu/madar/) corpus (Bouamor et al., 2018) included 12,000 sentences from a Basic Traveling Expression Corpus (BTEC) (Takezawa et al., 2007) and has been translated into French, MSA, and 25 Arabic dialects.

One of the earliest Arabic datasets created as an MSA Resource was the Penn Arabic Treebank (PATB) (Maamouri et al., 2004). It consisted of 350,000 words of newswire text and is available for a fee (https://catalog.ldc.upenn.edu/LDC2005T20). This dataset has been the main resource for some state-of-the-art systems and tools such as MADA (Habash, Rambow & Roth, 2009), and its successor MADAMIRA (Pasha et al., 2014), and YAMAMA (Khalifa, Zalmout & Habash, 2016b).

Of the Arabic dialects, as mentions before, the Egyptian dialect has had a wealth of attention; the earliest Egyptian corpuses are CALLHOME (Gadalla et al., 1997; Gamal et al., 2019), and MIKA (Ibrahim, Abdou & Gheith, 2015). Levantine Arabic has also received a lot of attention, as in the creation of the Levantine Arabic Treebank (LATB) (Maamouri et al., 2006), including 27,000 words in Jordanian Arabic. Some efforts were made for Tunisian (Masmoudi et al., 2014; Zribi et al., 2015), and Algerian (Smaıli et al., 2014). For Gulf Arabic, the Gumar corpus (Khalifa et al., 2016a) consists of 1,200 documents written in Gulf Arabic dialects from different forum novels available online (https://nyuad.nyu.edu/en/research/centers-labs-and-projects/computational-approaches-to-modeling-language-lab/resources.html). Using the Gumar corpus, a Morphological Corpus of the Emirati dialect was created (Khalifa et al., 2018), consisting of 200,000 Emirati Arabic dialect words, which is freely available (https://nyuad.nyu.edu/en/research/centers-labs-and-projects/computational-approaches-to-modeling-language-lab/resources.html). Table 1 shows more details about the Arabic corpora. As can be seen, besides the above-mentioned, most of which are freely available, a great majority mentioned in the related literature are not or involve strict procedures for gaining permission to reuse aggregated data. Additionally, most existing corpora do not offer free access.

**Table 1:** Comparison between different Arabic corpora.

**Corpus name**	**Ref.**	**Source**	**Size**	**Type**	**Online availability**
Al-Hayat Corpus	(De Roeck, 2002)	Al-Hayat newspaper articles	42,591	MSA	Available for a fee http://catalogue.elra.info/en-us/repository/browse/ELRA-W0030/
Arabic Lexicon for Business Reviews	Elhawary & Elfeky (2010)	Reviews	2,000 URLs	MSA	Not Available
AWATIF (a multi-genre corpus of Modern Standard Arabic)	Abdul-Mageed & Diab (2012)	Wikipedia Talk Pages (WTP), The Web forum (WF) and Part 1 V 3.0 (ATB1V3) of the Penn Arabic TreeBank (PATB)	2855 sentences from PATB, 5,342 sentences from WTP and 2,532 sentences from WF	MSA/Dialect	Not Available
The Arabic Opinion Holder Corpus	Elarnaoty, AbdelRahman & Fahmy (000)	News articles	1 MB news documents	MSA	Available at http://altec-center.org/
Large Arabic Book Review Corpus (LABR)	Aly & Atiya (2013)	Book reviews from GoodReads.com	63,257 book reviews	MSA/Dialect	Freely available at http://www.mohamedaly.info/datasets
Arabic Twitter Corpus	(Refaee & Rieser, 2014)	Twitter	8,868 tweets	Arabic dialect	Available via the ELRA repository.
An-Nahar Corpus	Eckart et al. (2014)	Newspaper text		MSA	Available for a fee https://catalog.elra.info/en-us/repository/browse/ELRA-W0027/
Tunisian Arabic Railway Interaction Corpus (TARIC)	(Masmoudi et al., 2014)	Dialogues in the Tunisian Railway Transport Network	4,662	Tunisian dialect	Not Available
DARDASHA	(Abdul-Mageed, Diab & Kübler, 2014)	Chat Maktoob (Egyptian website)	2,798	Arabic dialect	Not Available
TAGREED		Twitter	3,015	MSA/ Dialect	
TAHRIR		Wikipedia Talk pages	3,008	MSA	
MONTADA		Forums	3,097	MSA/ Dialect	
Hotel Reviews (HTL)	ElSahar & El-Beltagy (2014)	TripAdvisor.com	15,572	MSA/ Dialect	Not Available
Restaurant Reviews (RES)		Restaurant Reviews (RES) from Qaym.com	10,970	MSA/ Dialect	
Movie Reviews (MOV)		Movie Reviews (MOV) from Elcinemas.com	1,524	MSA/ Dialect	
Product Reviews (PROD)		Product Reviews (PROD) from Souq.com	4,272	MSA/ Dialect	
MIKA	(Ibrahim, Abdou & Gheith, 2015)	Twitter and different forum websites for TV shows, product and hotel reservation.	4,000 topics	MSA and Egyptian dialect	Not Available
Arabic Sentiment Tweets Dataset (ASTD)	(Nabil, Aly & Atiya, 2015)	Twitter	10,000 Egyptian dialect.	Egyptian dialect	Freely available at https://github.com/mahmoudnabil/ASTD
Health dataset	(Alayba et al., 2017)	Twitter	2026 tweets	Arabic dialect	Not Available
SUAR (Saudi corpus for NLP Applications and Resources)	(Al-Twairesh et al., 2018a; Al-Twairesh et al., 2018b)	Different social media sources such as Twitter, YouTube, Instagram and WhatsApp.	104,079 words	Saudi dialect	Not Available
Twitter Benchmark Dataset for Arabic Sentiment Analysis	(Gamal et al., 2019)	Twitter	151,000 sentences	MSA/ Egyptian dialect	Not Available

**Figure 1:**
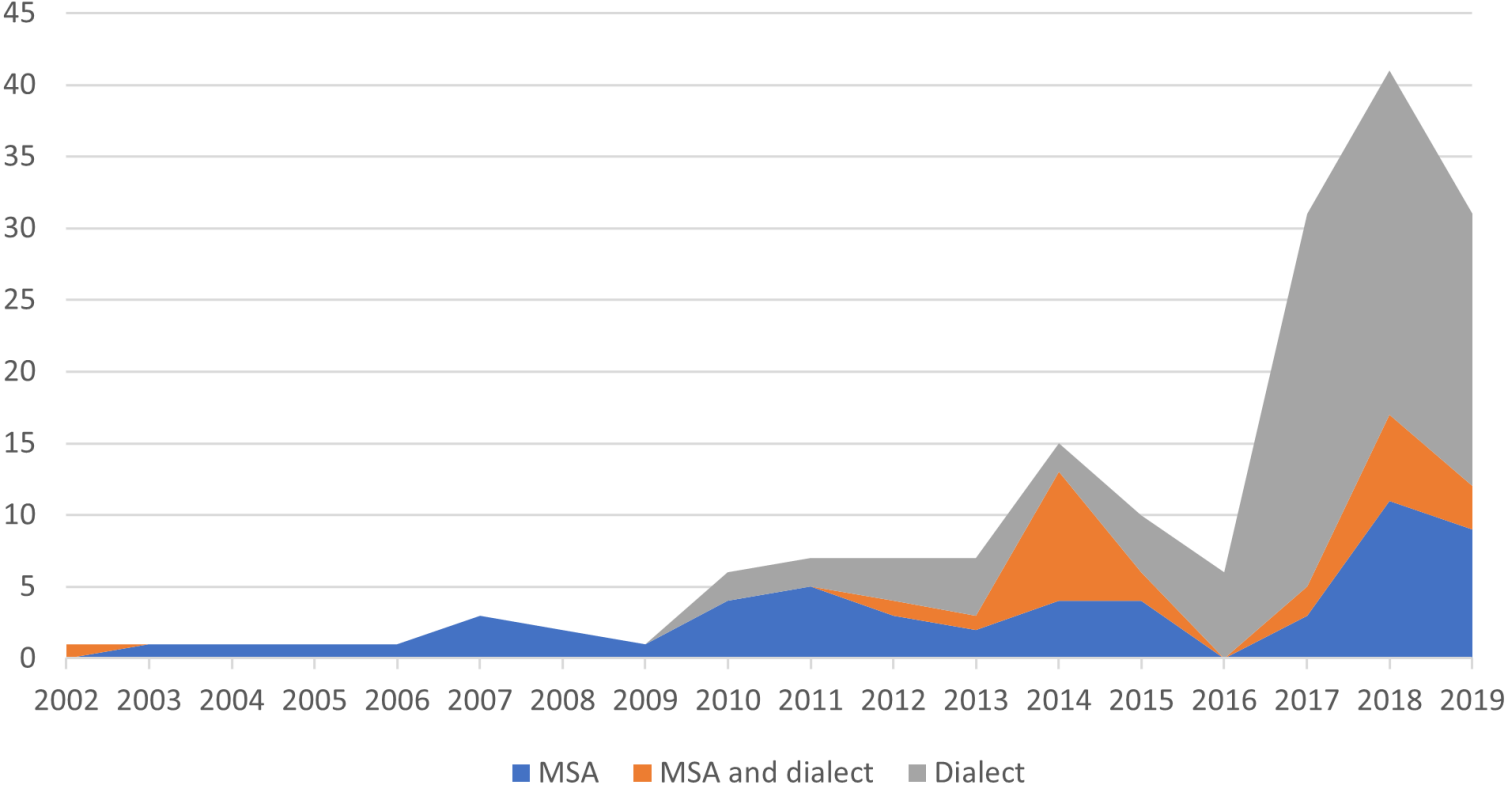
Percentage of Arabic corpora based on the type of corpus, from 2002 to 2019.

It is clear from Table 2 that the most-used source for the Saudi corpus is Twitter. Unfortunately, none of the Saudi corpus is available. In addition, some of them do not mention details about the annotation, which may pose a limitation for using these corpora. This paper aims to fill this gap by presenting the creation and annotation details about our GSC AraCust. In addition, we will make it freely available to the research community. Figure 1 illustrates the percentage of different Arabic corpus types. Interestingly, we found that since 2017, dialectal Arabic has been used in more corpora than MSA.

**Table 2:** Comparison between different Saudi dialect corpora for ASA.

**Corpus name**	**Ref.**	**Source**	**Size**	**Classification**	**Online availability**
AraSenti-Tweet Corpus of Arabic SA	(Al-Twairesh et al., 2017)	Twitter	17,573 tweets	Positive, negative, neutral, or mixed labels.	Not Available
Saudi Dialects Twitter Corpus (SDTC)	(Al-Thubaity et al., 2018)	Twitter	5,400 tweets	Positive, negative, neutral, objective, spam, or not sure.	Not Available
Sentiment corpus for Saudi dialect	Alqarafi et al. (2018)	Twitter	4,000 tweets	Positive or negative.	Not Available
Corpus for Sentiment Analysis	(Assiri, Emam & Al-Dossari, 2018)	Twitter	4,700 tweets		Not Available
Saudi public opinion	Azmi & Alzanin (2014)	Two Saudi newspapers	815 comments	Strongly positive, positive, negative, or strongly negative	Available upon request
Saudi corpus	Al-Harbi & Emam (2015)	Twitter	5,500 tweets	Positive, negative, or neutral	Not Available
Saudi corpus	Al-Rubaiee, Qiu & Li (2016)	Twitter	1,331 tweets	Positive, negative, or neutral	Not Available

## Data Collection

To build the dataset, we used Python to interact with Twitter’s search application programming interface (API) (Howard & Ruder, 2018) to fetch Arabic tweets based on certain search keys. The Python language and its libraries are one of the most flexible and popular approaches used in data analytics, especially for machine learning. To ensure pertinence to our target application, we started with hashtags related to the three largest Saudi telecom companies: the Saudi Telecom Company (STC), the Etihad Etisalat Company (Mobily), and Zain KSA, which dominate the market. As a result, we extracted the relevant top hashtags, as follows: #STC, #Mobily, #Zain, #,السعوديهand #, السعودية_ا which were used for the search. These initial seed terms were extracted based on the following Python function: *tags = API.trends. place ()* from the tweepy library. Additionally, we used the Twitter accounts of these companies as search keywords.

As the aim of this collection was to allow for a longitudinal, continuous study of telecom customers’ sentiments, we gathered data continuously from January to June 2017, mainly because this period includes customers’ reactions to the Saudi Communications and Information Technology Commission’s new index, which refers to complaints submitted to the authorities (Saudi InformationTechnology Commission, 2017). While seemingly a short period, it in fact generated the largest Arabic Telecom Twitter dataset for ASA. We were aware that we needed to account for the dataset subsequently reducing in size after spam and retweets were eliminated. The initial result obtained comprised 3.5 million tweets. After filtering and cleaning (based on location and time-zone and stratified random sampling; see below), the dataset was reduced to 795,500 Saudi tweets, which comprise the large AraCust dataset.

For our own further experimentations, in order to reduce computational costs and time in constructing our working AraCust corpus, we chose a sub-sample of Saudi tweets randomly from the dataset to prevent bias (Roberts & Torgerson, 1998). The principal notion behind the size reduction of the corpus was that the annotation process is manual, time-consuming, and costly. Specifically, to avoid bias in the sample, we applied the following steps: identify the population, specify the sample frame, and choose the right sample technique. As stated, the population in this study is STC, Mobily and Zain customer tweets. The sample frame is a Saudi tweet that describes the tweet author’s point of view regarding one of these companies. The probability sample technique is Simple Random Sample (SRS), applied stratified over the three sets (STC, Mobily, and Zain). The advantage of SRS is that all of the population has the same chance of being selected (Marshall, 1996). In addition, scholars have proven the efficiency of the random sampling technique for social media, because items that are repeated multiple times in the data set are likely to appear frequently in the sample as well (Kim et al., 2018; Gerlitz & Rieder, 2013).

The sample size decision was based on a pattern-extraction experiment using Network Overview, Discovery, and Exploration Node XL (Smith et al., 2009). Node XL is an add-in tool for Microsoft Excel used in social media analysis and visualization. Up to 2000 Arabic tweets were retrieved using the previously mentioned hashtags. Based on the findings of another study that 110 tweets per day are enough to capture customer sentiment (Assiri, Emam & Al-Dossari, 2018), we needed 20,000 tweets over 6 months. In addition, we found that the services provided by Saudi telecommunication companies most frequently mentioned in the customers’ tweets were: Internet speed, signal coverage, after-sales service, call centers, and fiber communication.

The size of our AraCust corpus of 20,000 Saudi tweets (Table 3) is in line with that of previous studies, which showed that datasets over 20,000 tweets are sufficient to produce state-of-the-art systems for Twitter Sentiment Analysis (SA) (Zhu, Kiritchenko & Mohammad, 2014; Mohammad, Kiritchenko & Zhu, 2013).

As the companies we targeted were from Saudi Arabia, we further filtered the tweets based on user location and time zone to identify Saudi tweets. Saudi Arabia ranks seventh in the world in the number of personal accounts on social media (Arab News, 2020). We found that many tweets do not have a location field set in the profile of the users who posted them. To resolve this issue, we used a list of city names, landmark names, city nicknames, etc., for Saudi Arabia, as additional labels for the user location of tweets, following Mubarak & Darwish (2014). Also following Mubarak and Darwish, we used a list from the GeoNames website (https://www.geonames.org/), a geographical database that includes 8 million place names for each country, which includes 25,253 place names for Saudi Arabia.

Finally, in the context of our data collection process from Twitter, it is worth mentioning that ethical concerns of using social media data have stirred an ongoing controversy in research communities in terms of confidentiality and privacy. The availability of social media data is thought to potentially expose social media users to risks. Although social media data is prominently public still, the emergence of profiling by business owners for business purposes has led to criticism and apprehension. Regarding our own study, on Twitter, users’ phone numbers and addresses are not made public, to provide some level of privacy. Additionally, in our current research, we further deleted any phone numbers or names that were included in the tweets themselves, for additional privacy. Finally, we collected only the tweet texts, time, and location, without collecting any other user-related information from them.

**Table 3:** Companies and the total number of unique tweets from each in AraCust.

**Company**	**Twitter Handle and hashtags**	**# of Unique Tweets**
STC	@STC_KSA, @STCcare, @STCLive	7,590
Mobily	@Mobily, @Mobily1100, @MobilyBusiness	6,460
Zain	@ZainKSA, @ZainHelpSA	5,950
Total		20,000

## Corpus Cleaning and Pre-Processing

To avoid noise in the corpus, cleaning was performed on the dataset. One way of cleaning is removing spam, thus any tweet with a Uniform Resource Locator (URL) was excluded, as in Al-Twairesh (2016) and Alayba et al. (2017), because most tweets in the dataset with a URL were news or spam. In addition, we excluded repetitive information, such as retweets, as recommended by Barbosa & Feng (2010) and Alayba et al. (2017). Moreover, non-Arabic tweets were excluded from the data set by filtering for Arabic language (lang: AR), because translation damages the classifier efficiency. Pre-processing was completed on the corpus using a Python script to remove unnecessary features in the tweets that might lower accuracy from the tweet corpus before applying classifiers, such as user mentions (@user), numbers, characters (such as + = ∼$) and stop words (such as “,”, “.”, “;”), as suggested by Refaee & Rieser (2014) and Al-Twairesh (2016). The tweet corpus was processed using the Natural Language Toolkit (NLTK) library in Python for normalization and tokenization. Although emoticons could arguably express sentiment, they were deleted, because prior research reported a classifier misunderstanding between the parentheses in the quote and in the emoticon (Al-Twairesh, 2016). In addition, importantly, as we dealt with Arabic tweets, Refaee & Rieser (2014) showed that retaining emoticons in classification decreased the performance of the classifier; they stated that this was due to the way Arabic sentences are written from right-to-left, which is reversed in emoticons.

Next, the words in the tweets were tokenized, which means that sentences were segmented into words for easier analysis, as in Al-Twairesh (2016) and Sun, Luo & Chen (2017). Finally, the tweets were normalized. For Arab text, normalization entails the unification of certain types of Arabic letters of different shapes, as in Al-Twairesh et al. (2017), i.e.:

• Replacing the Arabic letters “أ“, ”إ”, and “أ“ with bare *alif* “أ“.
• Replacing the letter ”ئ“, ”ئ“, and ”ئ“ with bare *ya* ”ئ“.
• Replacing the final “ة” with “ة”.
• If a word starts with “ء”, replacing it with “أ“.
• Replacing ”ؤ“ with ”ؤ“.

**Figure 2:**
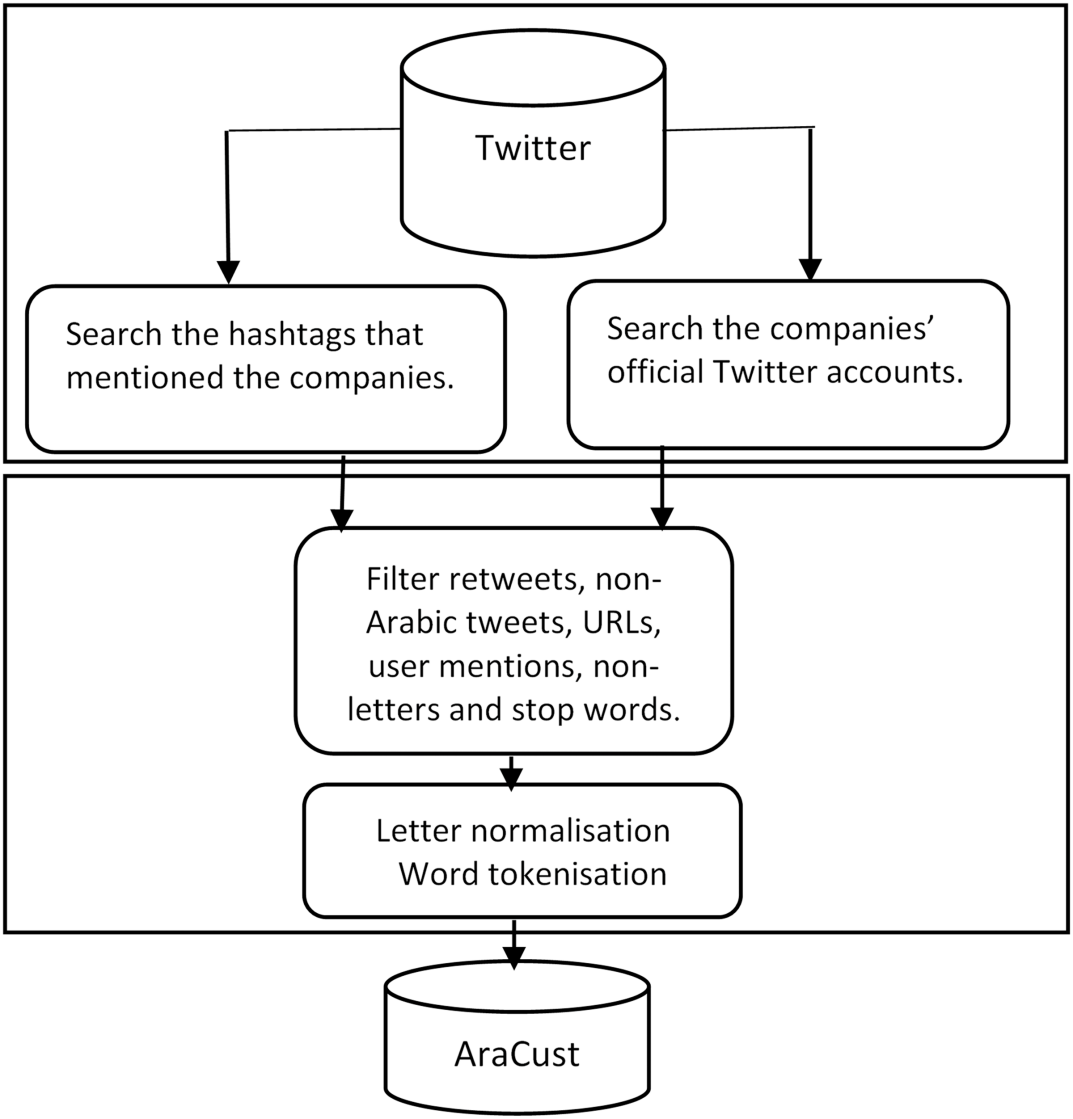
AraCust corpus collection, filtering and pre-processing.

As stemming algorithms do not perform well with DA words (Thelwall et al., 2011), they were not applied. The data collection, filtering, cleaning, and pre-processing steps are illustrated in Fig. 2. The subset before and after pre-processing is illustrated in Table 4. As shown in *m*, the emojis were deleted, and the prefix ““”ال “Al” was removed.

**Table 4:** Subset of the corpus before and after pre-processing.

**Tweet in Arabic**	**Label**	**Company**	**Tweet in English**	**Tweet after pre-processing**
@So2019So @STCcare الشركهغيري	Negative	STC	Change the Company	غيريشركه
@alrakoo @mmshibani @GOclub @Mobily฀اشكرك	Positive	Mobily	Thank you	اشكرك
@ZainKSA @CITC_withU يعوضنيعنالخسايرمين	Negative	Zain	Who will compensate me for the losses	مينيعوضنيعنخساير

## Exploratory Data Analysis

Before doing the sentiment analysis task, it is important to analyze the corpus. This includes the data types that we will deal with in the classification and prediction experiments, as well as the features that originate from the nature of the corpus, which may affect the model’s performance. Our data analysis involved many feature set analyses, from character-based to dictionary-based, and syntactic features (Soler-Company & Wanner, 2018). This exploratory data analysis was accomplished using character-based, sentence-based, and word-based features, to allow for processing at a variety of levels. The exploratory data analysis was completed using the NLTK library via a Python script.

**Figure 3:**
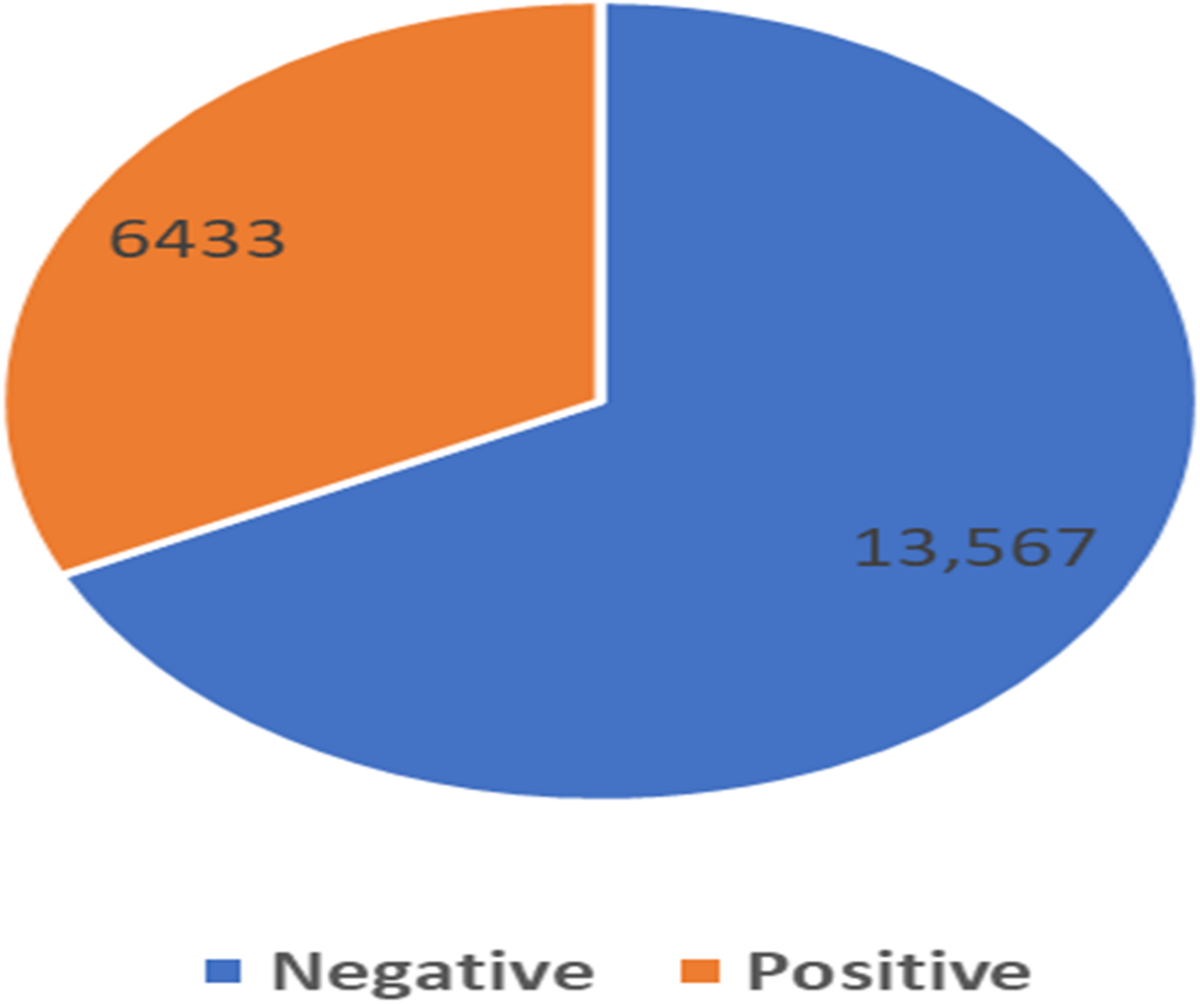
Distribution of negative and positive sentiment.

**Figure 4:**
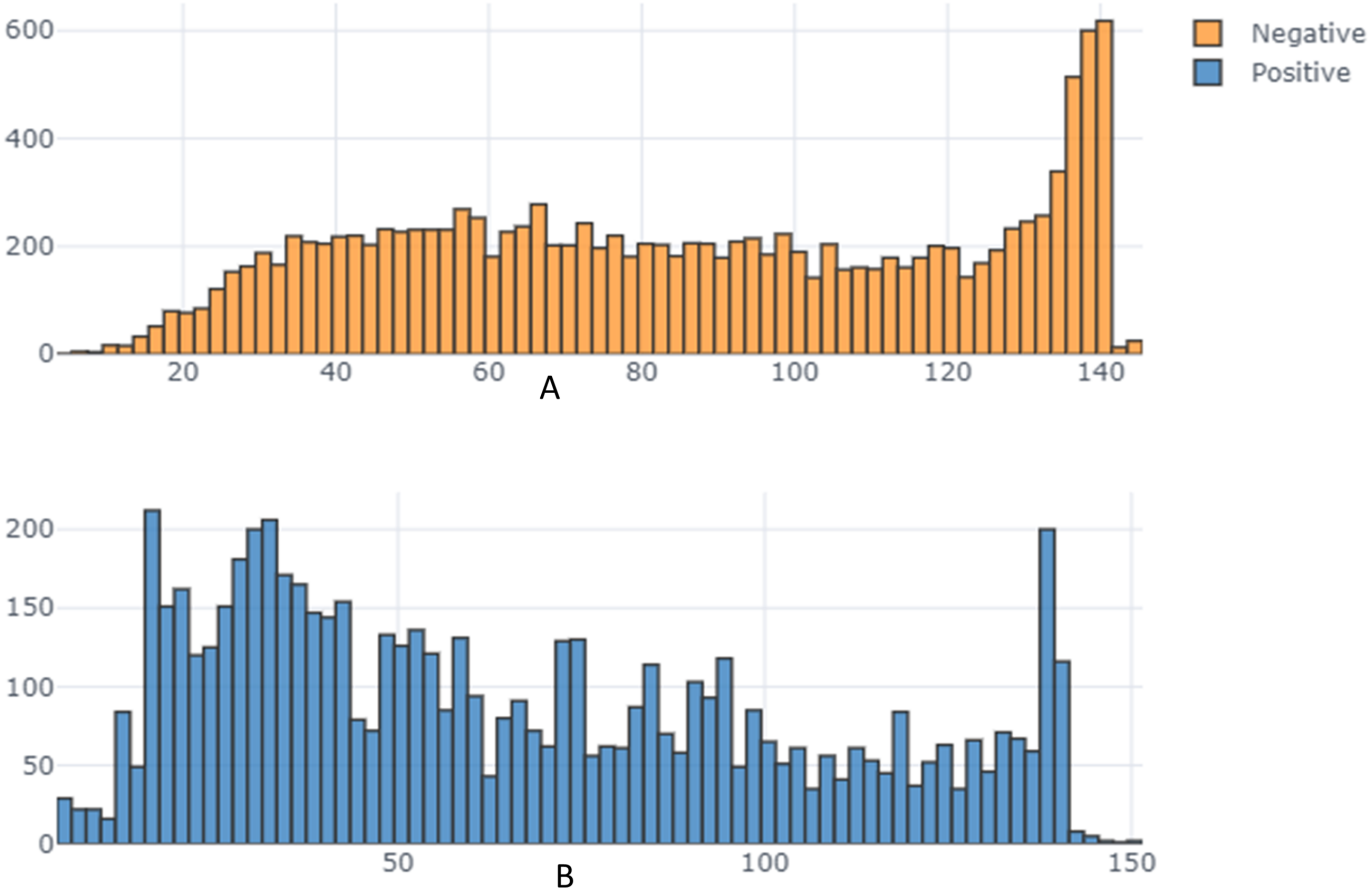
Tweet length distribution across sentiment.

**Figure 5:**
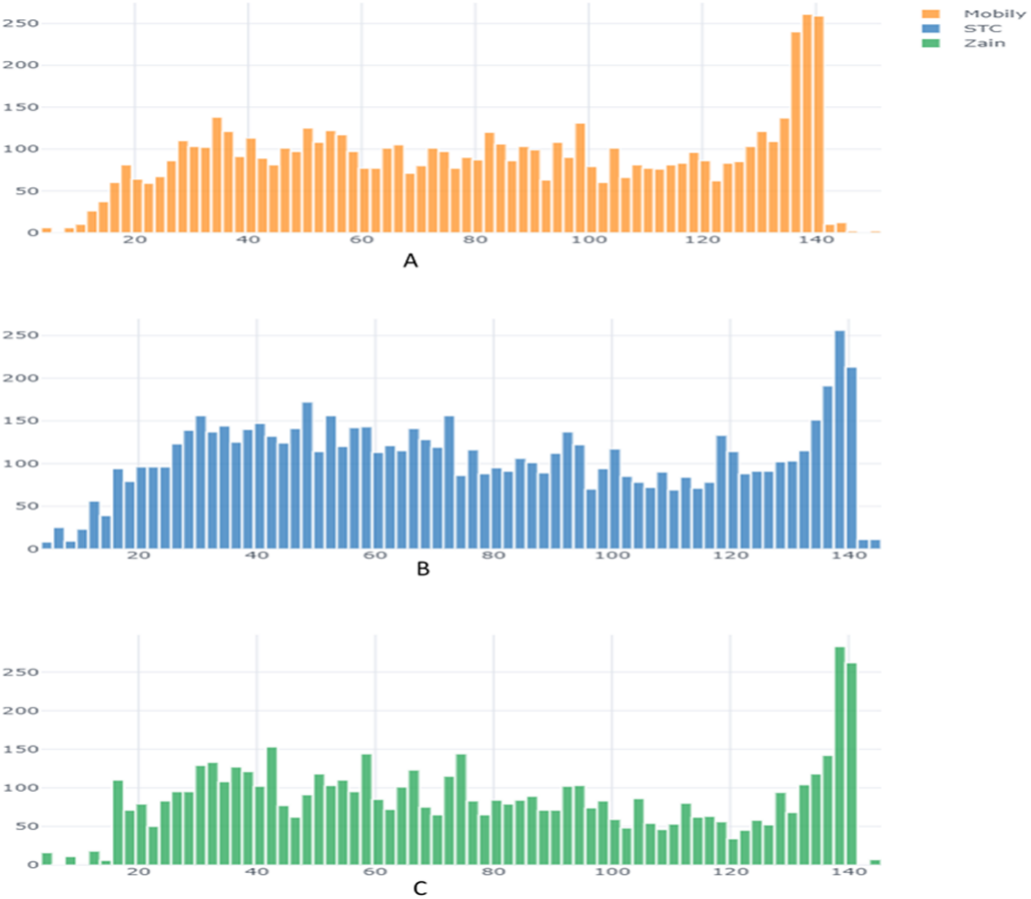
Tweet length distribution across companies.

From the exploratory data analysis, we observed first that there were more negative tweets than positive tweets for all three companies (see Table 5 and Fig. 3). We interpret this result as being due to all Arab countries having suffered difficult economic circumstances in the past few years; this result is in line with the findings by Refaee (2017) and Salameh, Mohammad & Kiritchenko (2015). Next, we analyzed the differences between the tweet length distribution across the sentiment to determine whether there was some potential correlation there and because prior research used the tweet-length feature as input to a machine learning classifier in SA research (Kiritchenko, Zhu & Mohammad, 2014; Al-Twairesh et al., 2018a) (Fig. 4). We observed that tweets tend to be longer when customers express a negative sentiment. In addition, interestingly, we found that STC customers had longer tweets overall than other companies’ customers (Fig. 5). These results guided us to use the All-Tweet Length feature in the classification task to estimate the impact of tweet length on the classifier’s performance.

**Table 5:** Companies and the total number of positive and negative tweets.

**Company**	**Negative**	**Positive**	**Total**
STC	5,065	2,525	7,590
Mobily	4,530	1,930	6,460
Zain	3,972	1,978	5,950
Total	13,567	6,433	20,000

The ten most frequent words in the corpus and their number of appearances in the corpus are given in Table 6. It appears from the table that there is a repeated use of the word “God,” but just from this information we do not know whether it was repeated in a negative or positive way. In addition, there was just one positive expression among these frequent words: “thank you” (which is one word in Arabic; see Table 6). The highest frequency was, naturally, for the word “Internet,” which potentially indicates the importance of this service; but likewise, we cannot tell at this stage if the reason for having “internet” among the most frequent words is positive or negative. To better understand the way these words are used, we first studied the context of usage by using the “most frequent” bigram to provide a more comprehensive view of the data.

**Table 6:** Most frequent words in the AraCust corpus.

**Word in Arabic**	**Frequency**	**Word in English**
نتا	1770	Internet
لله	1760	God
سلام	1363	Hello
والله	1179	Swear God
خاص	1315	Private
حسبي	637	Pray
عملاء	599	Customers
شكرا	560	Thank you
مشكلة	549	Problem
شريحة	515	Sim card

**Figure 6:**
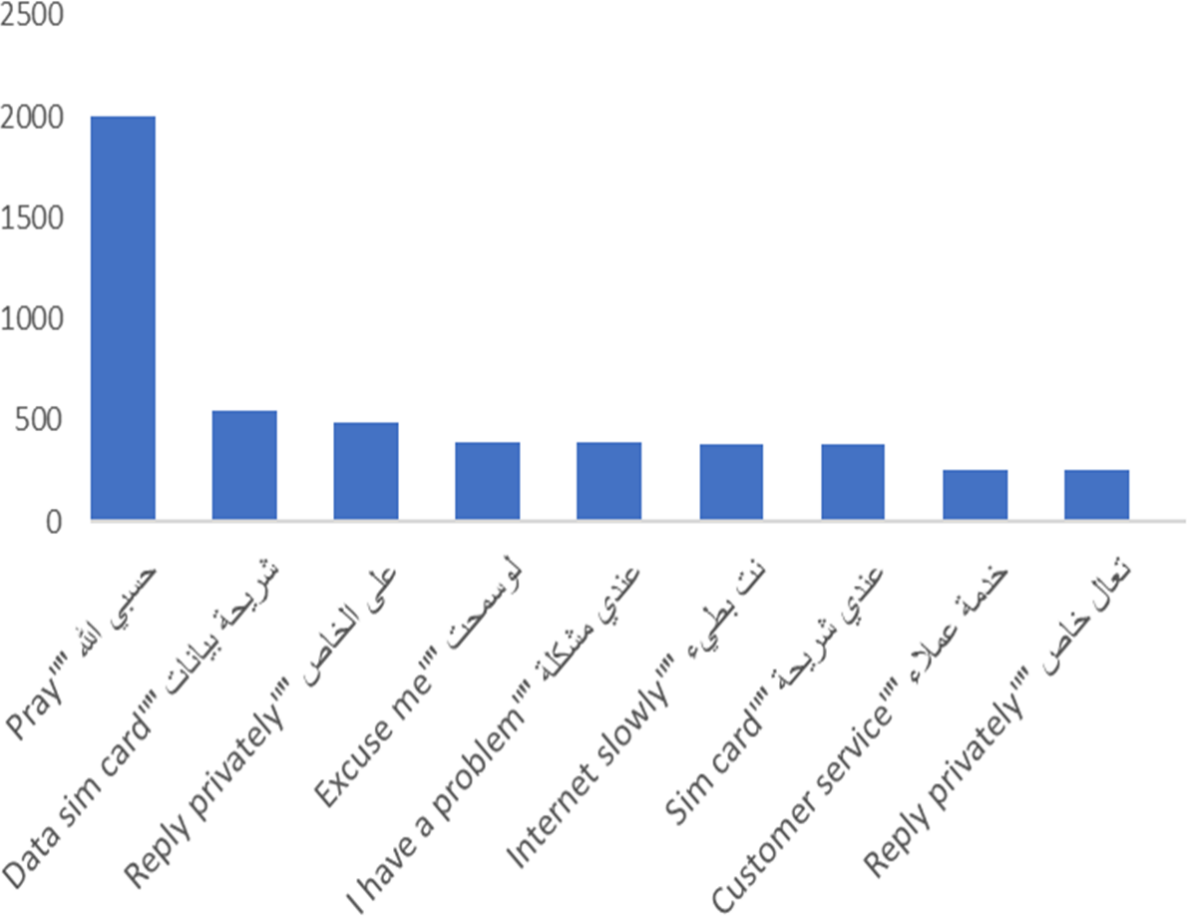
Most frequent Bigrams in the AraCust corpus.

The most frequent bigram on the corpus, as shown in Fig. 6, is “pray” (note that this is expressed as two words in Arabic); this is mainly used in a negative way, as explained below. Greetings are next in frequency, followed by “data sim card,” which we thought may to be due to a frequent problem source. We observed that internet service is described as slow, so most of the tweets that mentioned the internet are complaints, as shown below. Additionally, “customer service” is one of the most frequent bigrams in the corpus.

Next, we calculated the positive and negative rate for each word in the most frequent word chart to determine whether the word was used with a positive or negative sentiment. We calculated the positive rate *pr(t)* and negative rate *nr(t)* for the most frequent words (term *t*) in the corpus as follows (Table 7):

**Table 7:** Most frequent words in the AraCust corpus and their sentiment probability.

**Term in Arabic**	**Term in English**	**Negative**	**Positive**	**Total**	**Pos_rate**	**Neg_rate**
نت	Internet	975	795	1,770	0.44	0.55
الله	God	977	783	1,760	0.44	0.55
سلام	Hello	765	895	1,363	0.65	0.56
والله	Swear God	567	704	1,179	0.59	0.48
خاص	Private	656	659	1,315	0.50	0.49
حسبي	Pray	425	212	637	0.33	0.66
عملاء	Customers	413	186	599	0.31	0.68
شريحه	Sim card	271	289	560	0.51	0.48
مشكله	Problem	279	270	549	0.49	0.50
شكرا	Thank you	235	280	515	0.54	0.45

\begin{eqnarray*}pr(t)& = \frac{term\text{_}freq\text{_}df[t,`{positive}^{{^{\prime}}}]}{term\text{_}freq\text{_}df[t]} \end{eqnarray*}

\begin{eqnarray*}nr(t)& = \frac{term\text{_}freq\text{_}df[t,`{negative}^{{^{\prime}}}]}{term\text{_}freq\text{_}df[t]} \end{eqnarray*}

Where *term_freq_df [t, val]; val* ∈{*positive*, *negative*}; is the frequency of the word *t* as a word with valence (sentiment) *val* in the corpus:

\begin{eqnarray*}term\text{_}freq\text{_}df[t,val]=\sum _{tw\in C}^{n}\text{bool}1(\mathrm{tw},\mathrm{t},\mathrm{val}) \end{eqnarray*}

Where *tw* is a tweet in corpus *C*; and *bool1()* is a Boolean function:

\begin{eqnarray*}bool1(tw,t,val)= \left\{ \begin{array}{@{}ll@{}} \displaystyle 1, &\displaystyle valence(tw,t)=val\\ \displaystyle 0, &\displaystyle rest \end{array} \right. \end{eqnarray*}

With *valence(tw, t)* a function returning the sentiment of a word *t* in a tweet *tw* and term_freq_df [t] is the total frequency of the word *t* as both a positive and negative word in the corpus:

\begin{eqnarray*}term\text{_}freq\text{_}df[t]=\sum _{tw\in C}^{n}\text{bool}2(\mathrm{tw},\mathrm{t}) \end{eqnarray*}

Where *bool2()* is a Boolean function:

\begin{eqnarray*}bool2(tw,t)= \left\{ \begin{array}{@{}ll@{}} \displaystyle 1, &\displaystyle t\in tw\\ \displaystyle 0, &\displaystyle t\not\in tw \end{array} \right. \end{eqnarray*}

We found that “internet” is used as a negative word more than a positive word, as we discovered before. In addition, maybe surprisingly, the word “God” is used in negative tweets more than in positive ones. The words “Hello,” “Swear to God,” “private,” “sim card,” and “thank you” are used as positive words more than as negative words (contrary to our initial supposition that the frequency of “sim card” may indicate a problem). Moreover, we found the word “customers” used as a negative word more than a positive word.

These results led us to use the *Has Prayer feature* in the classification task; this feature allows us to evaluate whether the existence of a prayer in a tweet increases the classifier’s performance.

The feature set analysis is illustrated in Tables 8, 9 and 10. Character-based features (Table 8) reflect the existence of symbols, such as a minus sign, punctuation marks such as a comma, and numbers. The ratio was measured between the number of characters in a tweet and the number of characters overall.

**Table 8:** Character-based features.

**Character-based feature**	**Ratio**
Punctuation marks	8.0
Numbers	6.03
Symbol	0.0

**Table 9:** Sentence-based features.

**Sentence-based feature**	**Ratio**
Words per sentence	16.23
Sentence standard deviation	7.17
Range	30

**Table 10:** Word-based features.

**Word-based Feature**	**Ratio**
Word standard deviation	6.51
Word range	30
Chars per word	5.22
Vocabulary richness	1.0
Stop words	0.0
Proper nouns	0.11

Word-based features (Table 9) include word standard deviation, which was calculated using the standard deviation of word length, word range (the difference between the longest and shortest word), characters per word calculated by the mean number of characters for each word, and vocabulary richness, which is the count of various words.

Sentence-based features include the mean number of words for each sentence, the standard deviation of sentence length, and range (the difference between the longest and shortest sentence) (Table 10).

## Annotation

Before the SA, we needed to train the classifier and create a readable version for the machine using corpus annotation. Annotation is the process of assigning interpretative information to a document collection for mining use (Leech, 1993). Hinze et al. (2012) defined annotation as using predefined classes to mark the text, sentence, or words. Salameh, Mohammad & Kiritchenko (2015) defined annotation as providing the opinions and sentiments towards a target. There are different levels of corpus annotation. For example, sentiment annotation and syntactic annotation is the process of parsing every sentence in the corpus and labeling it with its structure, grammar, and part-of-speech (POS)—that is, labeling every word in the corpus with a corresponding appropriate POS label.

Several approaches used to annotate the corpus, including the manual approach, which depends on human labor, and the automatic approach, which uses an annotation tool.

*Gold Standard Corpora* (GSC) are an important requirement for the development of machine learning classifiers for natural language processing with efficiency; however, they are costly and time consuming and thus there are few GSCs available, especially for Arabic (Wissler et al., 2014).

The process of construction of the GSC is based on manual annotation by different experts who review the data individually, and then inter-annotator agreement is computed to confirm the quality (Wissler et al., 2014).

For sentiment annotation, several studies used three-way classification labels (positive, negative and neutral) to express sentiment orientation (Abbasi, Chen & Salem, 2008; Refaee & Rieser, 2014; Refaee & Rieser, 2016; Al-Twairesh, 2016). The output from the classification is based on the labels used in the annotation. In this research, we classified the corpora using binary classification (negative vs. positive) to predict customer satisfaction toward the telecom company, following many studies that used binary sentiment classification with Arabic text (Mourad & Darwish, 2013; Refaee & Rieser, 2016; Al-Twairesh, 2016; Abdul-Mageed, Diab & Kübler, 2014). Several prior studies have shown that binary classification is more accurate than other classifications (Refaee & Rieser, 2016; Al-Twairesh, 2016). Each sentiment label is a binary measure of customer satisfaction: “satisfied” and “unsatisfied.”

*Sarcasm* is a form of speech in which a person says something positive while he/she really means something negative, or vice versa (Liu, 2015). Sarcasm is notoriously hard to detect; in English, there are only a few studies on sarcasm detection using supervised and semi-supervised learning approaches (Liu, 2015). There have been no studies that have taken on sarcasm detection in ASA. Therefore, we asked the annotators to also label tweets with the presence of sarcasm, according to the sentiment they conveyed. This allowed us to be able to use sarcasm as a feature for machine learning classification, following Refaee & Rieser (2016). We thus opened the way for the first sarcasm-detection Arabic NLP work.

The corpora were divided into three corpora, based on the telecom company as the keyword (STC, Mobily, Zain). To ensure a high quality of the manual annotation process, clear guidelines were needed to maintain consistency between annotators (Al-Twairesh, 2016).

As recommended by Alayba et al. (2017) and Al-Twairesh (2016), three annotators were hired in this research to annotate our corpus. Our annotators, A1, A2, and A3, were all computer science graduates, native speakers of the Saudi dialect, and had prior annotation experience. The reason for choosing three annotators instead of the usual, and simpler, two, was to increase the quality of the resulting corpus by alleviating conflicts that could arise from discrepancies between only two annotators. Hence, if two annotators disagreed with respect to one tweet classification, we took a vote between all three annotators. In addition, Pustejovsky & Stubbs (2012) stated that more than two annotators is preferable.

To encourage a thorough examination of the tweets and high-quality results, the annotators were paid. Moreover, to ensure fair pay, in order to determine the annotators’ wages, we conducted a pilot study to calculate the average time they needed to annotate the tweets, as recommended by Al-Twairesh (2016). We provided the annotators with 110 tweets (Assiri, Emam & Al-Dossari, 2018) and the annotation guideline, and then calculated the average time that they needed for annotation. They took 33 min, 20 min, and 35 min to annotate the 110 tweets. Thus, the average time that they needed was 30 min to annotate 110 tweets. We then paid them to annotate the 20,000 tweets over the course of 2.5 months, two hours per day for five workdays per week.

**Figure 7:**
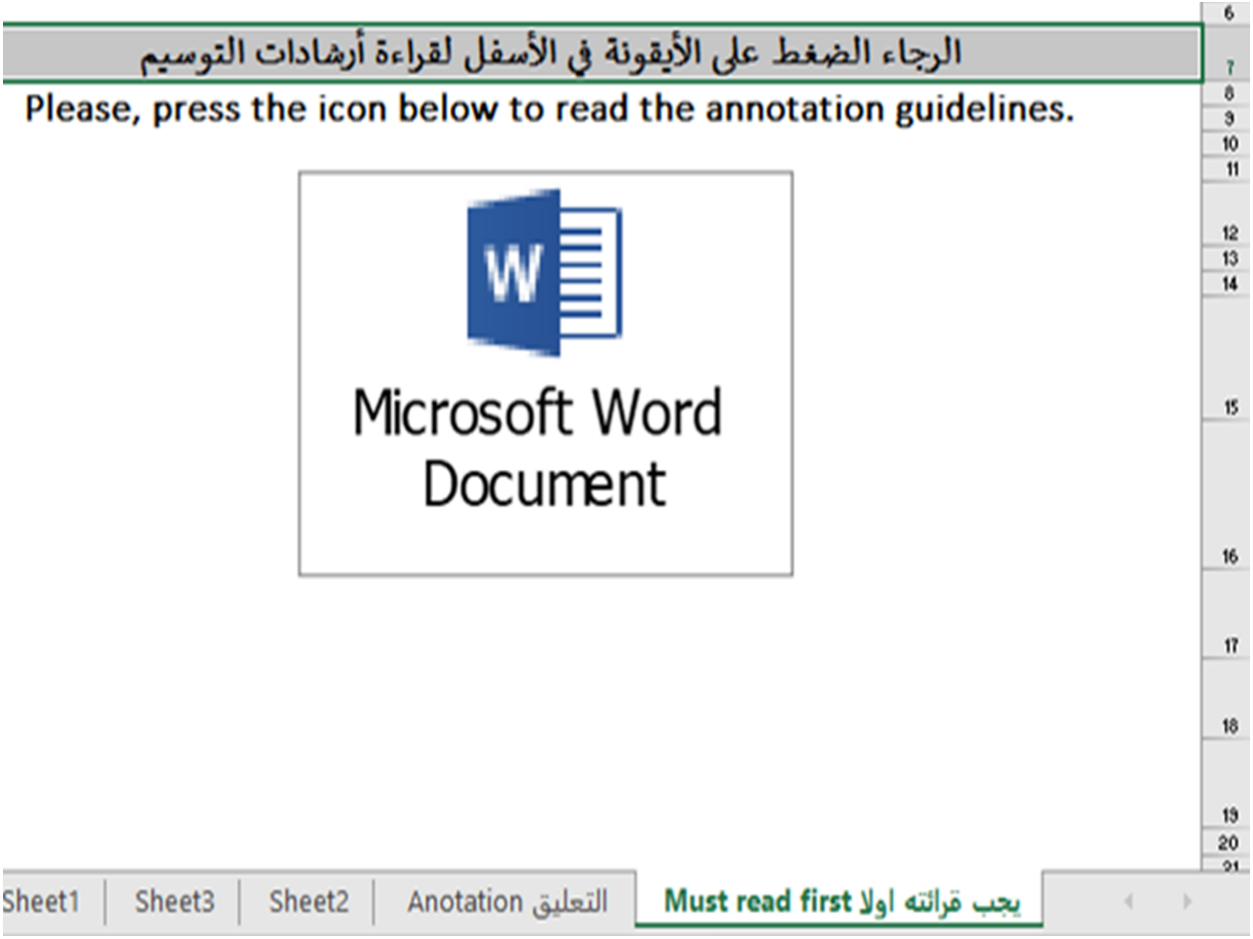
The included annotation guidelines in the XLSX file.

**Figure 8:**
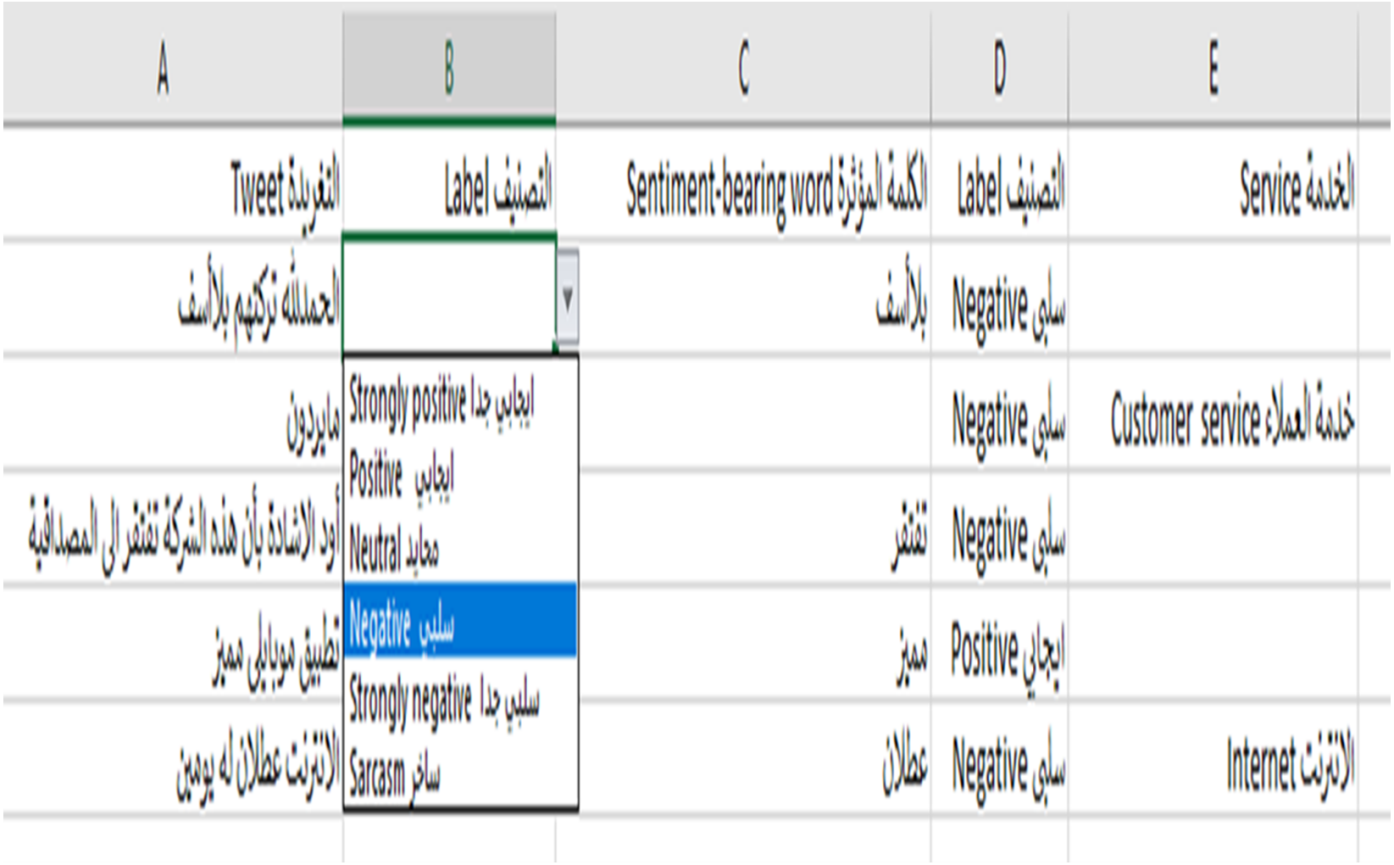
The annotation file.

Before we began the annotation process, the annotators were provided with annotation guidelines in both Arabic and English in a one-hour session; some of the annotation guidelines are shown in Table 11. We stored the annotations in an Excel file. The annotation guidelines were also included in the Excel file in case the annotator needed to read it (Fig. 7). As suggested by Pustejovsky & Stubbs (2012), we built an easy interface in the Excel file that has the tweets, an automatic list box of labels to avoid typing errors, the sentiment-bearing words, and the telecom services mentioned in the tweet, if found (Fig. 8).

To build a gold standard Arabic corpus, three rotations were used to annotate the corpus. As mentioned before, we divided the corpora into three based on the Telecom companies STC, Mobily, and Zain. They started the first rotation by annotating the STC corpus, then the Mobily corpus, followed by the Zain corpus. After the first rotation, we reviewed the annotators’ choices and discussed them with them before the new rotation started. After the second rotation, we calculated the similarity percentage between A1 and A2, A2 and A3, and A1 and A3 for the three corpora. At the third rotation, we asked the annotators to revise the labels for the corpus that have low similarity percentages. After the three rotations, the author revised the three annotation labels done by the annotators and compared their choices, using voting to make decisions. We found that 83% of the tweets were labeled with the same label by the A1 and A3, 75% of the tweets were classified with the same labels by A2 and A3, while 74% of the tweets were classified by A1 and A2 with the same labels.

**Table 11:** 

## Annotation Challenges

The annotators faced some challenges in the annotation process, similar to those experienced in prior research (Cambria et al., 2017), such as:

•  *Quoting and supplications*: It is difficult to define the sentiment of a tweet author whose tweet includes a quote or supplication, and to determine whether the author agrees with the sentiment of the quoted author. The annotators chose the sentiment that was expressed in the quote or in the supplication. Then, we checked the sentiment that they allocated. We did not ignore or remove the tweets with quotes or supplications, because the quotes/supplications were a form of expression of author sentiment.

•  *Sarcasm*: It is extremely hard to detect sarcasm in a tweet, because the explicit sentiment is different from the implicit sentiment. Nevertheless, as people are better at this than machines, annotation of tweets with this label is invaluable due to the difficulty of the sarcasm detection task (Rajadesingan, Zafarani & Liu, 2015). For that, we asked them to label a tweet accordingly if they could detect sarcasm in it.

•  *Defining the telecom services on the tweet:* The annotators indicated that not all of the tweets mentioned telecom services. This may be associated with the nature of the tweet, which is short. For this reason, we asked annotators to define the telecom services if they found them in the tweet.

•  *Absence of diacritics*: this makes the pronunciation of a word difficult, because without diacritical marks, some words have two possible meanings. For these, we asked the annotators to interpret the word in the context of its sentence.

## Inter-annotator Agreement

To identify the reliability of the annotation scheme, the inter-annotator agreement (IAA) was used. We used the similarity index as an early indicator of the annotators’ agreement. Fleiss’ Kappa (Davies & Fleiss, 1982) was used to measure consistency for the 5-way classification (Highly Positive, Positive, Neutral, Negative, Highly Negative) and for the binary classification (Positive, Negative), because there were more than two annotators (Davies & Fleiss, 1982; Fleiss, 1971).

The kappa **k Fleiss** (Fleiss, 1971) is defined as:

\begin{eqnarray*}k= \frac{\overline{P}-\overline{Pe}}{1-\overline{pe}} \end{eqnarray*}

Where $\overline{Pe}$ expresses the normalization of the agreement that is attainable randomly and $\overline{P}$ gives the normalized probability of agreement achieved by chance. If the annotators are in complete agreement, then *k = 1*. If there is no agreement among the annotators, then k <  0. The value we obtained was of 0.50 for 5-way classification and 0.60 for binary classification for the three annotators, which is a moderate level based on the level of acceptance (Landis & Koch, 1977). In addition, we checked for agreement two-by-two between A1 and A2, A1 and A3, and A2 and A3, and we took the average A (Table 12).

**Table 12:** Two-by-two agreement for binary classification between the three annotators.

**Annotators**	**k**
A1& A2	0.7
A2 & A3	0.74
A1 & A3	0.87
Avg A	0.77

## Evaluation of the Corpus

To evaluate our AraCust corpus, we applied a simple experiment using a supervised classifier to offer benchmark outcomes for forthcoming works. In addition, we applied the same supervised classifier on a publicly available Arabic dataset created from Twitter, ASTD (Nabil, Aly & Atiya, 2015), to compare the results of AraCust and ASTD; the details of these datasets are provided in Table 13. We used a Support Vector Machine (SVM), which has been used in Arabic sentiment analysis in recent research with high accuracy (Mubarak et al., 2020; Alayba et al., 2017; Bahassine et al., 2020). We used a binary classification (positive, negative) and eliminated tweets with different classification labels from the ASTD data set. We used a linear kernel with an SVM classifier, as some studies have stated that this is the best kernel for text classification (Mohammad, Kiritchenko & Zhu, 2013; Al-Twairesh et al., 2017; Refaee & Rieser, 2016). The AraCust and ASTD corpora were split into a training set and test set; additionally, 10-fold cross-validation was performed for both to obtain the best error estimate (James et al., 2013). For oversampling due to the dataset being biased towards negative tweets, we used the popular Synthetic Minority Over-Sampling Technique (SMOTE). The findings are in the test set, Table 14.

**Table 13:** Datasets used in the evaluation.

**Data Set**	**Positive tweets**	**Negative tweets**	**Total**
Aracust	6,433	13,567	20,000
ASTD	797	1,682	2,479

**Table 14:** Evaluation results of using the SVM on the datasets.

**Data Set**	**Positive**			**Negative**			**Total**	
	**Precision**	**Recall**	**F1**	**Precision**	**Recall**	**F1**	**F1 avg**	**Accuracy**
Aracust	93.0	76.0	83.6	91.0	98.0	94.4	89.0	91.0
ASTD	79.0	65.0	71.3	76.0	96.0	84.4	77.9	85.0

We analyzed the features term presence, term frequency (TF) (the frequency of each term within the document), and term frequency–inverse document frequency (TF–IDF) (the frequency of each word based on all records’ frequencies). We found that term presence is the best feature to use with binary classification, in line with what was found by Al-Twairesh et al. (2018a), which is that term presence is best for binary classification due to a lack of term repetition within a short text, such as a tweet. In addition, Forman (2003) stated that a term presence model can provide information such as term frequency for short texts. Pang & Lee (2008) noted that using term presence leads to better performance than using term frequency. The results in Table 14 show that our dataset AraCust outperforms the ASTD result. Further research may also investigate using deep learning algorithms on our newly created GSC AraCust dataset.

## Study Validation

This study used a sentiment analysis on GSC AraCust to measure customer satisfaction. To validate the proposed approach, we developed a simple questionnaire of two questions. The questionnaire is oriented towards the customers whose tweets were mined, to compare the predicted customer satisfaction using the proposed approach with actual customer satisfaction using the questionnaire (Table 15*)*.

**Table 15:** Percentage of predicted customers satisfaction vs. actual customer’s satisfaction.

**Company**	**Predicted customer’s satisfaction**	**Actual customer’s satisfaction**
**STC**	40.01%	20.1%
**Mobily**	39.00%	22.89%
**Zain**	34.06%	22.91%

**Figure 9:**
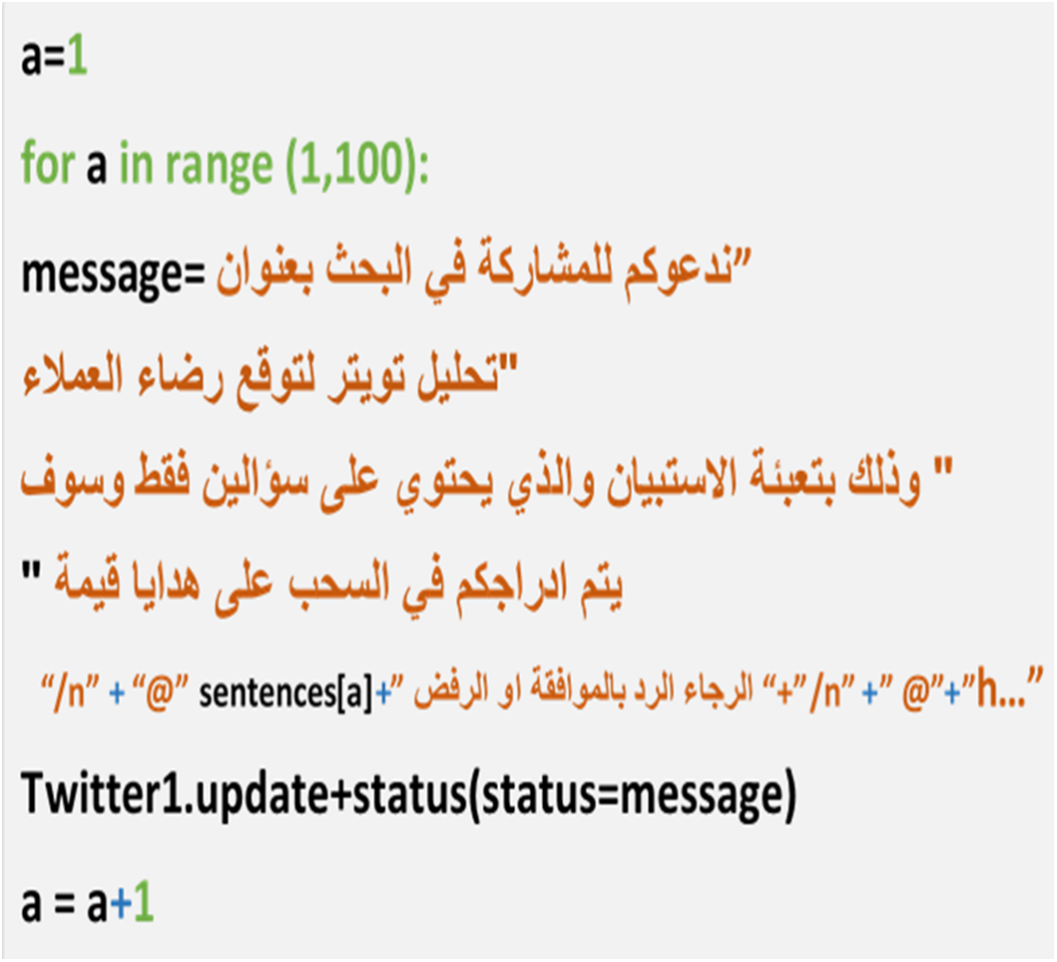
Snapshot from the Python code for tweets generator.

**Figure 10:**
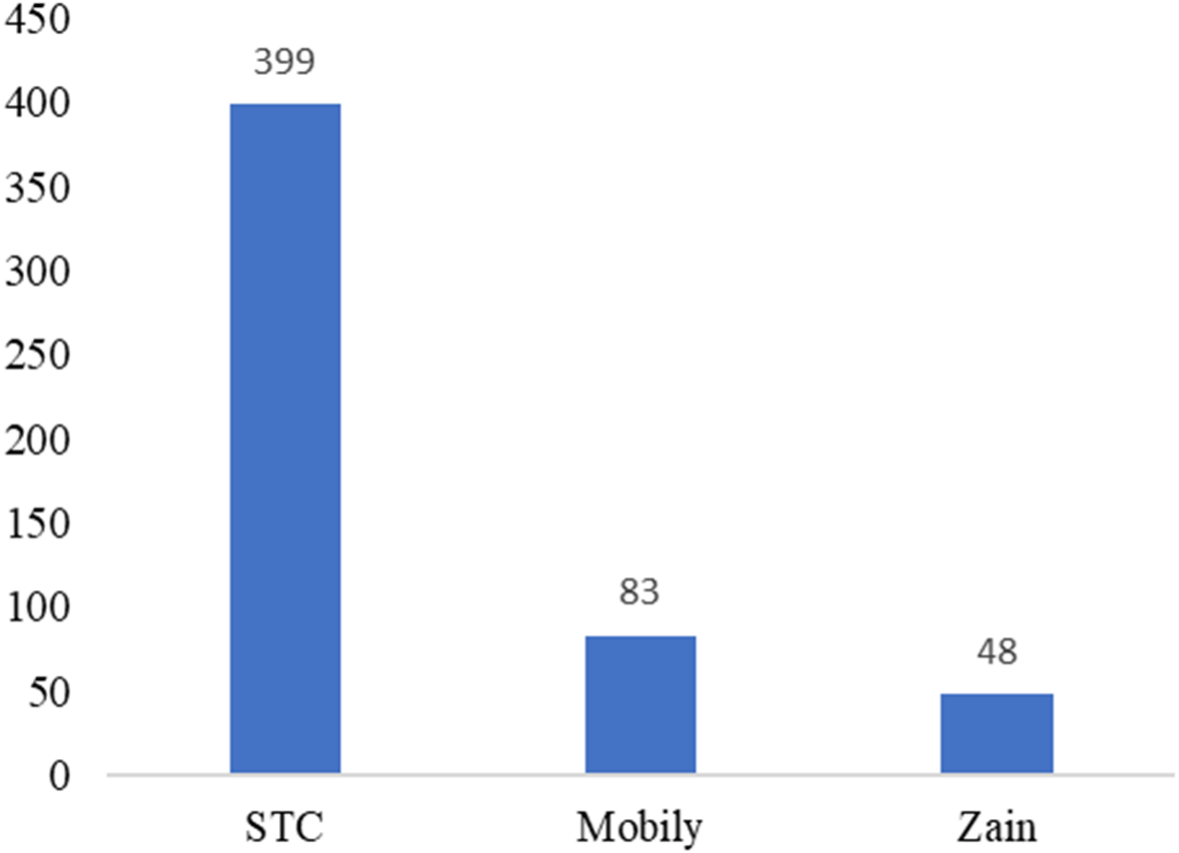
Number of participants based on telecom companies.

We made an automatic tweet generator in Python (the tweet has a link to the questionnaire) to all 20,000 users whose tweets we had previously mined, but the respondents totaled just 200. The tweet generator was created using a code in Python for sending tweets that have two things (the link to the questionnaire and mentions to the Twitter accounts of participants). To save time, the code completed this procedure automatically (Fig. 9). The questionnaire was built in Google Forms because it is easy to build and distribute. The questions were: “What is your telecom company?” and “Define your satisfaction toward your company (satisfied, unsatisfied).” We received 530 responses. The sample was distributed between customers of the three companies, as shown in Fig. 10.

**Figure 11:**
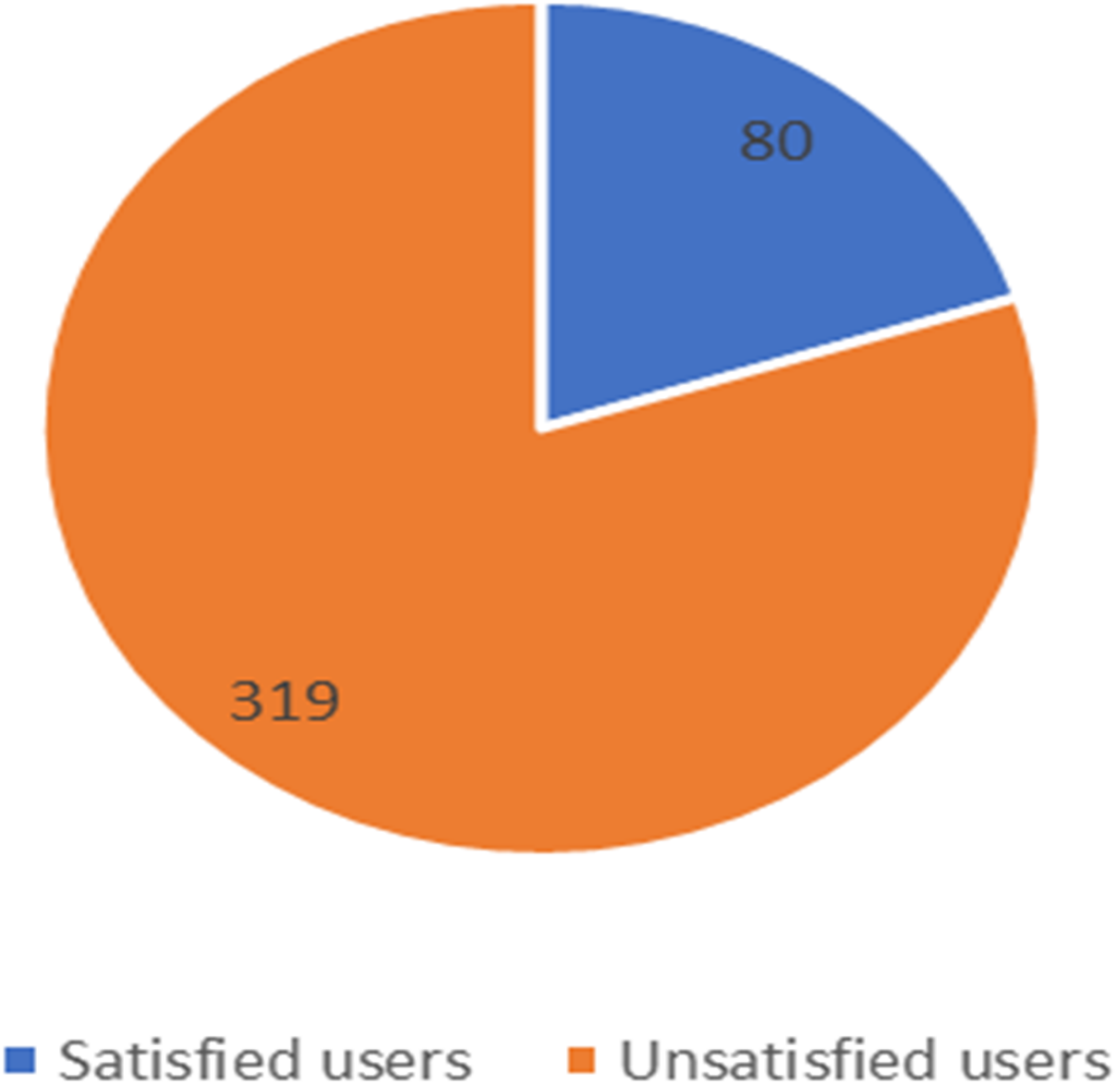
Number of satisfied and unsatisfied users for STC company.

**Figure 12:**
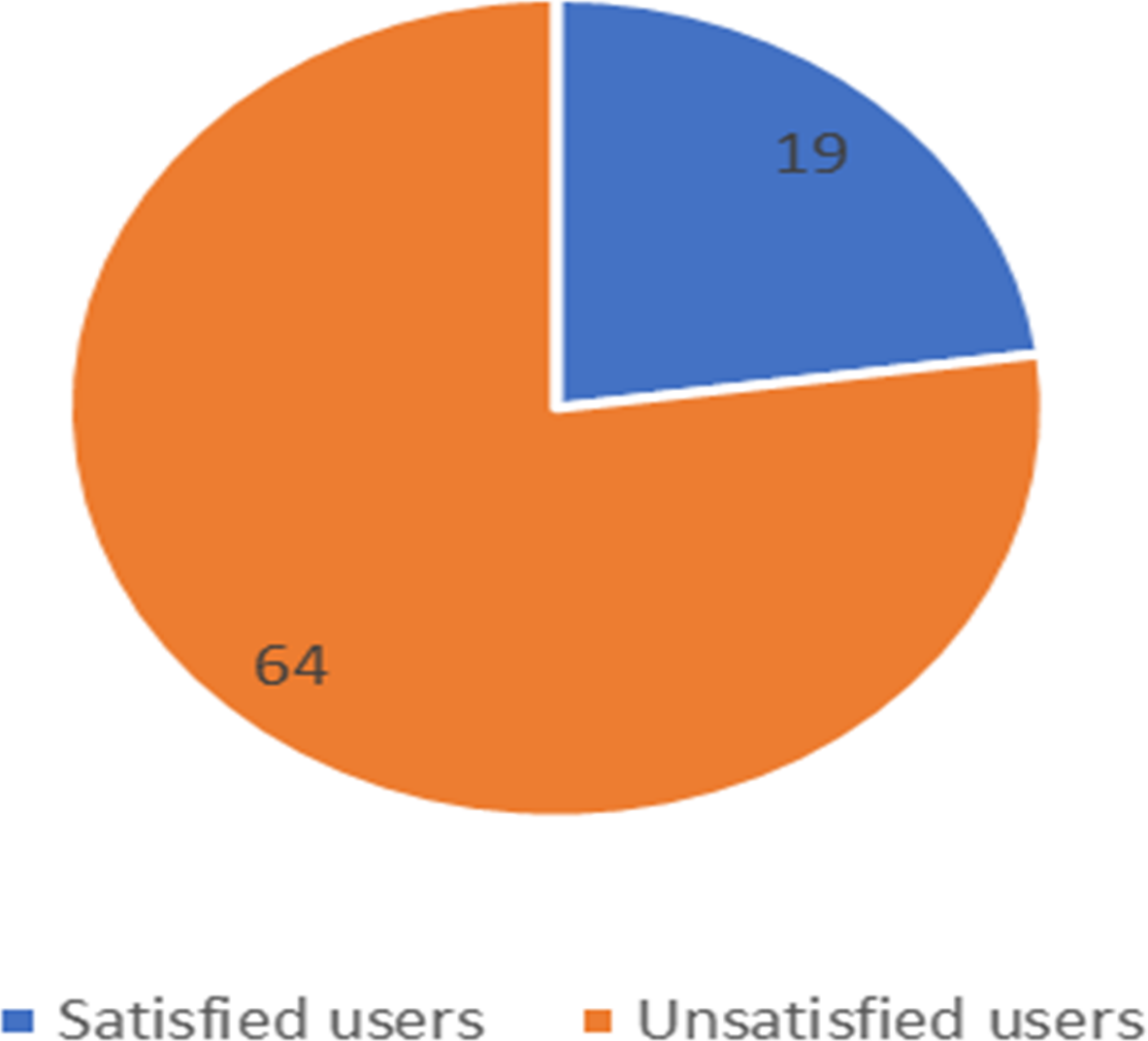
Number of satisfied and unsatisfied users for Mobily company.

**Figure 13:**
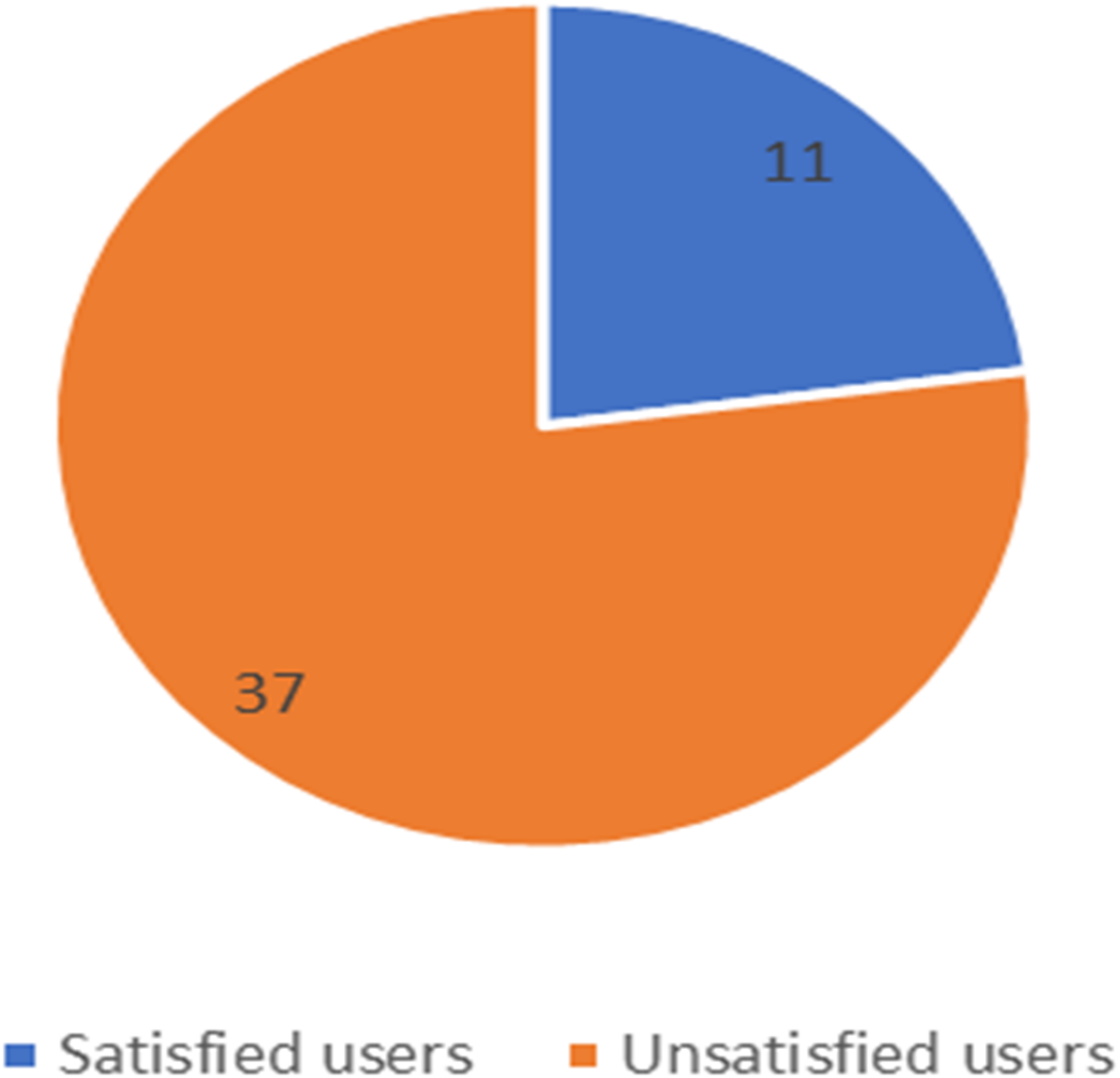
Number of satisfied and unsatisfied users for Zain company.

The unbalanced numbers of participants between the three companies reflects the real distribution of the users of the Saudi telecom companies. The number of unsatisfied and satisfied users for STC is shown in Fig. 11, for Mobily in Fig. 12, and for Zain in Fig. 13.

Table 15 shows that the proposed approach achieved the goal of predicting customer satisfaction of telecom companies based on the Twitter analysis.

These results can provide insights for the decision-makers in these companies regarding the percentage of customer satisfaction and help to improve the services provided by these companies. These results should encourage decision-makers to consider using Twitter analyses for measuring customer satisfaction and to include it as a new method for evaluating their marketing strategies.

## Conclusion

This study set out to fill gaps in the literature by proposing the largest gold-standard corpus of Saudi tweets created for ASA. It is freely available to the research community. This paper described in detail the creation and pre-processing of our GSC AraCust, explained the annotation steps that were adopted in creating AraCust, and described features of the corpus, which consists of 20,000 Saudi tweets. A baseline experiment was applied on AraCust to offer benchmark results for forthcoming works. Additionally, a baseline experiment was applied to ASTD to compare the results with AraCust. The results show that AraCust is superior to ASTD. Further generalization of the dataset use can look into other aspects of the communications of customers of the three majors Saudi providers of telecom services—serving, for instance, a total of 41.63 million subscribers who use mobile voice communication services. Furthermore, we have informed the telecom service companies of our results at every step of our investigation, and these results, dataset, and overall methodology may be used in the future to improve their services for their customers.

##  Supplemental Information

10.7717/peerj-cs.510/supp-1 Supplemental Information 1 Python code for corpus preprocessing and exploratory data analysis

10.7717/peerj-cs.510/supp-2 Supplemental Information 2 Telecom Corpus

## Additional Information and Declarations

### Data Availability

The following information was supplied regarding data availability: The code and data are available in the Supplemental Files.
